# Deciphering CO_2_ flux and fluorescent DOM origins in the carbon cycle of Kaptai Lake

**DOI:** 10.1371/journal.pone.0334646

**Published:** 2025-11-14

**Authors:** Osman Miah, Rhidi Barma, Md. Anamul Hassan, Mashura Shammi, Shafi M. Tareq

**Affiliations:** Hydrobiogeochemistry and Pollution Control Laboratory, Department of Environmental Sciences, Jahangirnagar University, Dhaka, Bangladesh; National Research and Innovation Agency, INDONESIA

## Abstract

This study aimed to assess the current water quality, identify the sources of fluorescent dissolved organic matter (fDOM), and quantify the CO_2_ flux from Kaptai Lake surface water. A water quality multiparameter analyzer, a membrane-enclosed pCO_2_ sensor, and a weather monitoring device were deployed to continuously record data over 48 hours to observe daily and spatial shifts. All measured water quality parameters remained within the acceptable limits set by the Department of Environment (DoE). The three-dimensional excitation-emission matrix (3D-EEM) analysis identified distinct fluorophores at peak A (Ex/Em = 245/404 nm), peak M (Ex/Em = 310/404 nm), peak T (Ex/Em = 280/338–346 nm), and peak Tuv (Ex/Em = 230/338–350 nm). Parallel factor analysis (PARAFAC) modelling further resolved these into protein-like components and fulvic-like substances, specifically C-like and M-like fluorophores, indicating the presence of both microbial and terrestrial sources. Spatial distribution patterns of fDOM intensity suggest variability driven by localized pollution sources across the lake. Optical indices further indicated that the fDOM components were predominantly biologically derived, characterized by low aromaticity, lower molecular weight and size, and were largely influenced by microbial degradation processes. Diurnal monitoring of partial pressure of CO_2_ (pCO_2_) in the lake water revealed values ranging from 577 to 1045 µatm. Correspondingly, the CO_2_ flux (FCO_2_) varied between 45 and 56 mmol CO_2_ m ⁻ ^2^ d ⁻ ^1^. The positive average FCO₂ indicates that the lake acts as a net source of CO_2_ to the regional atmosphere. Higher pCO_2_ levels are linked to lower dissolved oxygen and increased protein-like DOM that fuels microbial respiration, while humic-like DOM helps stabilize carbon by limiting CO_2_ release.

## Introduction

Kaptai Lake is Bangladesh largest man-made lake, renowned for its stunning natural beauty and vital role in hydroelectric power generation, fisheries, and tourism, which was created by damming the Karnaphuli River in the Chattogram Hill Tracts. The construction of the H-shaped Kaptai Lake commenced in 1961 with the primary objective of hydroelectric power generation [1]. This development marked a significant milestone in the advancement of renewable hydroelectric energy in Bangladesh during the early 1960s. Beyond its role in energy production, Kaptai Lake remains vital for sustaining freshwater ecosystems, supporting local livelihoods, and providing water resources to the surrounding Chattogram region. Intensified anthropogenic activities have led to increased pollutant loads, placing significant stress on water resources and contributing to climate change. These impacts also alter the composition and fluxes of dissolved organic matter (DOM), thereby polluting aquatic ecosystems [2–4]. Dissolved organic matter (DOM) encompasses a complex and heterogeneous mixture of carbon-based compounds pervasive across aquatic environments [5]. Its primary natural sources include algal exudates, plant and soil organic matter (SOM) decomposition, zooplankton excretions, and microbial processing of organic material [6,7]. In addition, DOM composition is increasingly influenced by anthropogenic inputs such as biomass burning, agricultural runoff, fossil fuel combustion, and industrial discharges [8]. The diversity and reactivity of DOM reflect this blend of natural and human-derived sources. Lakes, in particular, are rich reservoirs of DOM, playing a pivotal role in aquatic ecosystems by modulating light penetration, nutrient cycling, and microbial activity [5]. Given their capacity to store and transform organic matter, lakes significantly influence the biogeochemical dynamics of surrounding landscapes, with their impact often scaling with surface area and depth [9].

Understanding the behavior and sources of fluorescent dissolved organic matter (fDOM) is essential for tracing pollutant dynamics in aquatic systems [10]. Fluorescence spectroscopy, particularly three-dimensional excitation-emission matrix (3D-EEM) combined with parallel factor analysis (PARAFAC), has emerged as a powerful approach for characterizing the origin, composition, and biogeochemical functions of dissolved organic matter (DOM) in diverse aquatic environments [11–13]. 3D-EEM enables high-resolution mapping of fluorescence intensity across excitation and emission wavelengths, while PARAFAC facilitates the decomposition of complex fluorescence matrices into independent, chemically meaningful components [14]. To ensure both the reliability and interpretability of the PARAFAC model, we employed a comprehensive set of validation techniques. These methods followed the recommendations of [12] as well as the community guidelines provided by Open. These included split-half analysis to confirm model stability to assess component robustness and verify component similarity with established spectral signatures. This rigorous validation framework enhances confidence in the identification of fDOM components and their environmental relevance. Recent studies in Bangladesh have employed EEM-PARAFAC approaches to assess fDOM in rivers and lakes, revealing widespread anthropogenic contamination of surface waters [15–19]. Our study builds upon this foundation by integrating validated PARAFAC modelling with fluorescence spectroscopy to robustly characterize fDOM dynamics in freshwater systems.

Carbon dioxide (CO_2_) is a potent greenhouse gas and a key component of the global biogeochemical carbon cycle [20]. While anthropogenic sources such as fossil fuel combustion, industrial activities, and transportation are widely recognized as dominant drivers of atmospheric CO_2_ enrichment [21], natural processes also play a significant role in CO_2_ emissions. Within inland waters, CO_2_ concentrations are modulated by a complex interplay of factors, including photochemical degradation [22], microbial respiration [23], oxygen availability [24], photosynthetic uptake [25], hydrological inputs from precipitation and runoff [26], temperature fluctuations [27], and the metabolic activity of phytoplankton and algae [28]. Quantifying the flux of CO_2_ (FCO_2_) between aquatic systems and the atmosphere is critical for advancing our understanding of the global carbon budget [29]. Recent estimates suggest that FCO_2_ emissions from inland waters range between 0.75 and 3.88 Pg C yr⁻^1^ substantially exceeding fluxes from marine (2.51 Pg C yr⁻^1^) and even terrestrial (3.24 Pg C yr⁻^1^) ecosystems [16]. Despite their relatively small areal extent, lakes contribute disproportionately to global FCO_2_ due to their high surface-specific CO_2_ emission rates [30]. Unlike oceans, which primarily serve as CO_2_ sinks, freshwater systems can act as either sources or sinks depending on local biogeochemical conditions [29]. A global survey of 1,835 lakes revealed that approximately 87% were supersaturated with CO_2_ relative to atmospheric equilibrium [29,31]. The dynamics of CO_2_ fluxes in inland waters are influenced by both natural variability and anthropogenic disturbance. Since the onset of the industrial era, land use change, sewage discharge, soil erosion, agricultural water abstraction, dam construction, petroleum contamination, and climate change have collectively driven an estimated increase in inland water FCO_2_ emissions by approximately 1.0 Pg C yr⁻^1^ [32].

This study investigated the diurnal and spatial variability of fluorescent dissolved organic matter (fDOM) components in lake water, alongside continuous 24-hour monitoring of partial pressure of carbon dioxide (pCO_2_), to elucidate the lake’s carbon dynamics and determine its role as a net source or sink of atmospheric CO_2_. Given the lake’s substantial surface area, quantifying the magnitude of CO_2_ flux (FCO_2_) and identifying the origins of its organic matter are critical for evaluating its response to climatic drivers and environmental pressures in the surrounding region. Also, Depending on their light availability during the day and the microbiological activity during the night, the diurnal release of CO_2_ exhibits distinct patterns. Although PARAFAC modeling has been widely applied to characterize DOM in aquatic environments, research on subtropical artificial reservoirs such as Kaptai Lake, Bangladesh, remains limited. The relationships between CO_2_ flux dynamics and fluorescent DOM sources in these systems are poorly understood. This study addresses this gap by integrating high-frequency CO_2_ flux measurements with PARAFAC-derived DOM characterization, providing new insights into carbon cycling in subtropical reservoirs. Given the limited spatial coverage, broader seasonal and spatial sampling will be essential for a more comprehensive understanding of DOM dynamics.

## Materials and methods

### Study area

**Fig 1:**
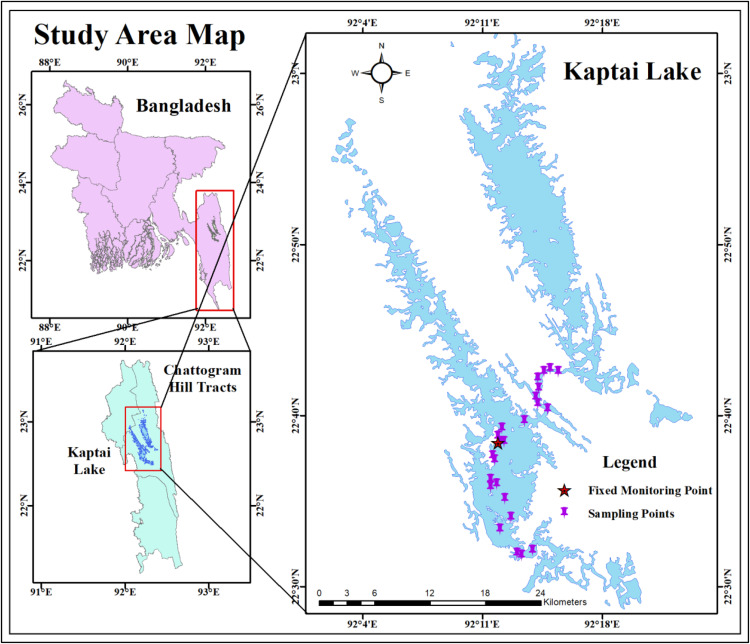
Monitoring station and sampling locations in the Kaptai Lake, Bangladesh.

This study was conducted at Kaptai Lake, the largest artificial freshwater reservoir in southeastern Bangladesh, located in the Rangamati District (Fig 1). Formed by damming the Karnaphuli River, Kaptai Lake spans a water surface area of approximately 583 km^2^, with a total area of 688 km^2^ and a catchment basin covering 11,200 km^2^ [1]. The lake has an average depth of 30 meters and connects to the Karnaphuli River on its eastern side. Topographically, Kaptai Lake is divided into two distinct arms by intervening highlands and hilly terrain: the northeastern arm, which receives inflow primarily from the Kasalong tributary, and the southwestern arm, which functions as the main reservoir and is fed by the Chengi tributary [33]. The lake’s elevation above means sea level ranges from 350 to 1000 meters, reflecting the varied topography of the region [34]. Only water samples were collected for physicochemical and biochemical analyses, without disturbing aquatic biodiversity, protected species, or restricted zones. As Kaptai Lake is a public water body and tourist spot under the different governmental authorities in Bangladesh, with no restrictions on basic water sampling, no specific permits were required.

### Field measurement and data collection

In mid-May 2022, a 48-hour continuous monitoring campaign was conducted at a fixed site on Kaptai Lake (22°38’25.5“ N, 92°11’54.6” E) in Rangamati, Bangladesh, to assess diurnal variations in water quality, physicochemical parameters, and air–water partial pressure of CO₂ (pCO₂). Surface water samples (n = 24) were collected every 2 hours from 20 cm depth using acid-washed 10 mL vials. Samples for fluorescence spectroscopy were immediately filtered through pre-combusted 0.45 µm GF/F filters and stored at 4 °C before transport to the Hydrobiogeochemistry and Pollution Control Laboratory, Jahangirnagar University. An additional 20 spatial samples (1 L each) were collected at 1-km intervals across the lake to evaluate fDOM distribution (Fig 1). In situ measurements of pH, TDS, DO, EC, turbidity, temperature, and fDOM were obtained using a YSI EXO2 multiparameter sonde. High-frequency monitoring of pCO_2_ in surface water (20 cm depth) and ambient air (1 m above the surface) was conducted using a diffusion-based CO_2_ sensor and CR1000X datalogger, with temperature and pressure corrections applied [35]. Air temperature and relative humidity were continuously recorded using a HOBO USB Micro Station (H21-USB). All sensors recorded data at 10-second intervals, which were subsequently averaging to 1-minute intervals to improve clarity of presentation.

### EEM-PARAFAC modelling

Fluorescence excitation–emission matrices (EEMs) of the water samples were recorded using a HITACHI F-4600 fluorescence spectrometer (Japan) at room temperature. Fluorescence spectra were acquired in three-dimensional mode using a 700 V xenon lamp at a scanning speed of 1200 nm min^−1^. The excitation wavelengths ranged from 200 to 400 nm (5 nm intervals), while emission wavelengths spanned 250–500 nm (1 nm intervals). To correct for instrumental and sample-related artifacts, EEMs were pre-processed by subtracting Milli-Q water blanks and adjusting for inner filter effects using absorbance-based correction factors. Fluorescence intensities were normalized to the Raman peak of Milli-Q water and reported in Raman Units (RU). Negative fluorescence values were replaced with zero to prevent computational artifacts during modeling [12]. Parallel Factor Analysis (PARAFAC) was conducted on the processed EEMs using the DOMFluor Toolbox (v1.7). To ensure model reliability, we performed a split-half validation with random initialization to confirm component robustness [12]. The validated components were then cross-referenced with the OpenFluor database to identify similarities with previously reported fluorophores from other freshwater systems, providing further external validation and comparability [36]. Two PARAFAC models were developed: one based on 24 diurnal samples to assess temporal dynamics, and another using 20 spatially distributed samples to evaluate geographic variability in fDOM composition across Kaptai Lake. A detailed discussion of component similarity to DOM signatures from other aquatic environments is along with model diagnostics and validation results.

### Air–Water CO_2_ flux calculation

The flux of CO_2_ across the air–water interface (FCO_2_) was calculated using the following mass transfer equation:


$${FCO}_{\text{2}}=\Delta CO\text{2}\times k$$ (Eq. 1)

where ΔCO_2_ represents the concentration gradient between water and air, and k is the gas transfer velocity [37–39]. ΔCO_2_ was derived from the difference between the partial pressure of CO_2_ in surface water (pCO_2__water) and ambient air (pCO_2__air), multiplied by the temperature-dependent solubility coefficient (*K_h_*), as shown in Equation 2:


$${\Delta CO}_{\text{2}}=K_{h}\times (pCO\text{2}\_water-pCO\text{2}\_air)$$ (Eq. 2)

The solubility coefficient K_h_ (mol·L ⁻ ¹·atm ⁻ ¹) was calculated as a function of water temperature (*T*, in °C) using the empirical relationship:


$$K_{h}={\text{1}\text{0}}^{\left(–\text{1}.\text{1}\text{1}+\text{0}.\text{0}\text{1}\text{6}\times T–\text{0}.\text{0}\text{0}\text{0}\text{0}\text{7}\times T²\right)}$$ (Eq. 3)

Water temperature was measured in real time using a YSI EXO2 multiparameter sonde. Both pCO_2__water and pCO_2__air were recorded on-site using a diffusion-based CO_2_ sensor connected to a CR1000X datalogger. The gas transfer velocity k was obtained from previously published values relevant to similar freshwater systems [38].

### Dissolved Organic Carbon (DOC) and optical indices analysis

Ultraviolet-visible (UV-Vis) absorbance spectra of water samples were recorded using a SPECORD 210 Plus UV-Vis spectrophotometer (Analytik Jena, Germany) over a wavelength range of 200–800 nm at a scan rate of 50 nm s^−1^, using Milli-Q water as a reference. Because of laboratory limitations in conducting instrumental analysis with a total organic carbon (TOC) analyzer, DOC concentrations were estimated using a two-wavelength absorbance model based on UV absorbance at 254 and 365 nm [40]. Specific ultraviolet absorbance at 254 nm (SUVA₍_254_₎) was calculated as SUVA = A₂₅₄/DOC, providing insight into the aromaticity of DOM [41]. The spectral slope ratio (SR = S₂₇₅–₂₉₅/S₃₅₀–₄₀₀) and the absorbance ratio E₂₅₀E₃₆₅ were calculated from absorption coefficients at relevant wavelengths to infer DOM molecular weight and aromaticity [42,43].

Three fluorescence-based indices were used to further characterize DOM sources and composition: **Fluorescence Index (FI)** was calculated from emission intensities at 450 nm and 500 nm under 370 nm excitation (FI = f₄₅₀/f₅₀₀). FI values near 1.3 suggest terrestrial (allochthonous) DOM, while values near 1.8 indicate microbial (autochthonous) sources [14,44]. **Humification Index (HIX)** was determined from excitation at 254 nm as the ratio of the integrated emission intensities from 435–480 nm (H) to 300–345 nm (L): HIX = H/L. Higher HIX values (>10) indicate highly humified terrestrial DOM, while values <4 suggest lower humification and more recent microbial input [45]. **Biological Index (BIX)** was calculated using excitation at 310 nm and emission at 380 and 430 nm (BIX = f₃₈₀/f₄₃₀). BIX values >1.0 are typically associated with fresh autochthonous production, whereas values <0.6 imply aged or degraded material [46]. All optical index calculations adhered to standard methodological protocols. To ensure reliability, a comprehensive interpretation of FI, HIX, and BIX values is provided in the Results and Discussion section, referencing thresholds widely reported in literature to support source attribution of DOM.

### Quality assurance, data validation, and statistical analysis

An extended 48-hour field campaign was conducted to capture a full 24-hour diurnal cycle of water quality and CO_2_ data within the sensor’s equilibrium period. To adjust for the response time of the diffusion-type CO_2_ sensor in flowing water conditions, approximately three hours of unstable data were excluded from the dataset. Prior to each monitoring session, all field sensors were calibrated using certified standard solution of known concentrations. Before analyzing samples in the lab, cuvette cells used for fluorescence spectroscopy were thoroughly rinsed with Milli-Q water and a mild 5% HNO_3_ acid solution to eliminate contamination. Appropriate blanks and calibration standards were included in every run to ensure accuracy of the fluorescence and UV-Vis measurements. Data processing and statistical analysis were carried out using MATLAB R2022b and Microsoft Excel (Office Professional Plus 2016). Standard deviations and errors were calculated using Microsoft Excel. Correlation analyses among variables were performed using OriginPro 2021. To visualize spatial patterns and sampling points, ArcGIS version 10.8 was employed to generate detailed mapping outputs. This multi-software workflow ensured both analytical precision and reproducibility across the different stages of field and lab-based data handling.

## Results and discussion

### Assessment of physicochemical properties

The statistical analysis of key physicochemical parameters (Table 1) from the lake water samples (n = 1440) in a fixed position offers a comprehensive insight into its current ecological state in comparison with national standards and other lake systems in Bangladesh. The average water temperature ranged from 27.83 °C to 32.13 °C (31.34 ± 0.72) °C, slightly exceeding the Department of Environment’s (DoE, 2023) upper threshold of 30°C. This marginal rise could be attributed to heat influence, reduced canopy cover, or ongoing climate variability, Kaptai Lake shows a seasonal temperature range of 21.1 °C to 33.8 °C, similar to Dhanmondi Lake (18–32 °C) and Hatirjheel Lake (22.1–31.5 °C), reflecting the influence of shallow depth and climate [47,48]. In contrast, the deeper Lake Bogakain exhibits more stable temperatures (22.4–27.0 °C) due to thermal stratification [49]. Also, Rahman et al. 2014 found 29.10 °C to 32.80 °C in Kaptai Lake [50]. Despite the thermal elevation, the dissolved oxygen (DO) levels remained consistently high (6.91 ± 0.19 mg/L), which is encouraging from an ecological standpoint and a similar result was found 7.6–8.2 mg/L [51]. Such levels are typically associated with limited organic loading and moderate aeration. Dissolved oxygen levels in Kaptai Lake ranged from 4.6 to 8.9 mg/L, while Lake Bogakain exhibited more stable and moderately high DO levels (5.8–7.1 mg/L) due to its depth and limited anthropogenic disturbance [47,49].

**Table 1:** Physicochemical Parameters of Kaptai Lake.

Parameter	Mean Value	Standard Deviation	Degrees of Freedom (df)	t-Statistic	p-Value	F-Statistic	F-Critical (0.05 level)	Water quality standard (DoE, 2023)
Water Temp. (^0^C)	31.34	0.72	1439	1651.76	<0.0001	0.5184	3.8415	20-30
DO (mg/L)	6.91	0.19	1439	1380.08	<0.0001	0.0361	3.8415	≥ 5
pH	7.07	0.11	1439	2438.98	<0.0001	0.0121	3.8415	6.5–8.5
EC (µS/cm)	125.82	0.86	1439	1236.93	<0.0001	0.7396	3.8415	≤ 1200
TDS (mg/L)	72.92	0.88	1439	1471.87	<0.0001	0.7744	3.8415	≤ 1000
Turbidity (NTU)	9.29	0.39	1439	903.92	<0.0001	0.1521	3.8415	≤ 10
Salinity (PSU)	0.05	0.001	1439	1897.37	<0.0001	0.0001	3.8415	≤ 0.5
fDOM (RFU)	3.36	0.60	1439	212.51	<0.0001	0.36	3.8415	NA

The pH of the lake water was nearly neutral (7.07 ± 0.11), well within the acceptable range (6.5–8.5), indicating balanced carbonate buffering and minimal acidification risks. But previous studies found slightly more pH from 7.46 to 7.76 [52]. The electrical conductivity (EC) averaged 125.82 ± 0.86 µS/cm, reflecting low ionic content and indicating that the lake is not significantly impacted by mineral or wastewater inflows. Similarly, total dissolved solids (TDS) were measured at 72.92 ± 0.88 mg/L. The previous research finds a low TDS value of 47.8–58.6 mg/L [51]. Turbidity was slightly elevated (9.29 ± 0.39 NTU) but remained within the DoE limit (≤10 NTU), possibly influenced by occasional sediment disturbance or surface runoff during rainfall. The lake was also confirmed to be freshwater, with salinity at just 0.05 ± 0.001 PSU. An interesting observation was the presence of fluorescent dissolved organic matter (fDOM) at a mean of 3.36 ± 0.60 RFU. Although no specific national guideline exists for fDOM.

All parameters showed statistically significant deviations from their null hypothesis values (p < 0.0001), indicating real differences in the data distribution rather than random variation. Additionally, low F-statistics compared to the critical value (F = 3.8415) suggest homogeneity of variance across the sample population. Overall, the lake appears to be in relatively good ecological condition, with values aligning closely with national standards and comparable clean-water bodies in Bangladesh. However, the slightly elevated temperature and turbidity levels warrant regular monitoring to detect any emerging trends.

### Dissolved organic carbon and optical properties of DOM

Surface water samples from Kaptai Lake exhibited a mean dissolved organic carbon (DOC) concentration of 0.81 ± 0.12 mg L^−1^, which is comparable to levels reported for urban lakes in Dhaka City (0.803 ± 0.001 mg L^−1^; [15]). This value also falls within the range observed in coastal and riverine systems of Bangladesh, including the Sundarbans Mangrove Forest (0.9 ± 0.1 mg L^−1^) and the Brahmaputra River during the dry season (1.03 ± 0.23 mg L^−1^; [17]), as well as in nearshore waters of the Bay of Bengal (0.78–0.94 mg L^−1^; [53]). These comparable concentrations underscore a regional coherence in DOC inputs and transformation across diverse aquatic systems of South Asia.

**Fig 2:**
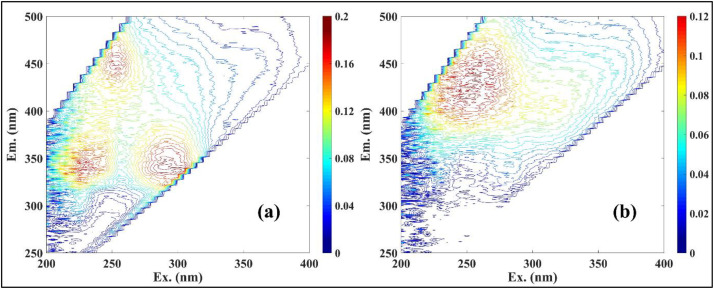
Representative 3D-EEM spectra of water samples collected from Kaptai Lake: (a) fixed monitoring point and (b) spatial distribution (mid lake point).

To investigate DOM compositional characteristics, excitation-emission matrix (EEM) fluorescence spectroscopy was conducted on 48 lake water samples. Three prominent fluorescence peaks, peak T (Ex/Em = 260–285/310–380 nm), peak Tuv (Ex/Em = 220–240/280–360 nm), and peak A (Ex/Em = 260–280/380–460 nm) were consistently observed (Fig 2a). Peaks T and Tuv are characteristic of protein-like fluorophores (tryptophan and tyrosine-like), associated with recent microbial activity and labile, autochthonous DOM inputs [54], while peak A is indicative of humic-like substances derived from terrestrial or microbial degradation pathways [55,56]. Fluorescence intensity exhibited marked diel variation, likely reflecting coupled microbial and photochemical dynamics. Peak T ranged from 0.06 to 0.56 RU (0.19 ± 0.04), peak Tuv from 0.08 to 0.85 RU (0.28 ± 0.07), and peak A from 0.11 to 0.58 RU (0.18 ± 0.04) (S1 Fig.). Maximum intensities were recorded during the dusk period (6–8 PM), consistent with enhanced microbial processing or photo-altered DOM release following daytime solar exposure. In contrast, peak T and Tuv intensities were lowest in the late morning (10 AM), whereas peak A reached a minimum around midnight (2 AM), pointing to distinct temporal controls on DOM reactivity and transformation.

Spatial heterogeneity in DOM composition was further elucidated using parallel factor analysis (PARAFAC). One major component revealed dual fluorophore signatures, peak A at Ex/Em = 240/405 nm and peak M at Ex/Em = 300/405 nm, both associated with fulvic acid-like DOM fractions. These fluorophores are typically attributed to semi-labile or refractory DOM pools, often resulting from microbial reworking or terrestrial organic matter leaching into aquatic systems [13]. The intensities of peaks A and M ranged from 0.07–0.23 RU (0.09 ± 0.008) and 0.04–0.13 RU (0.06 ± 0.005), respectively (S2 Fig.), reflecting moderate spatial variability in DOM source and lability.

Collectively, these optical and temporal signatures point to a dynamic DOM regime in Kaptai Lake, shaped by microbial production, photochemical alteration, and variable allochthonous inputs. The prominence of protein-like components underscores the contribution of freshly produced, bioavailable DOM to the system’s carbon pool, whereas fulvic-like signals highlight slower-turnover fractions with implications for longer-term carbon storage and lake-atmosphere CO_2_ flux regulation.

### PARAFAC component analysis

**Fig 3:**
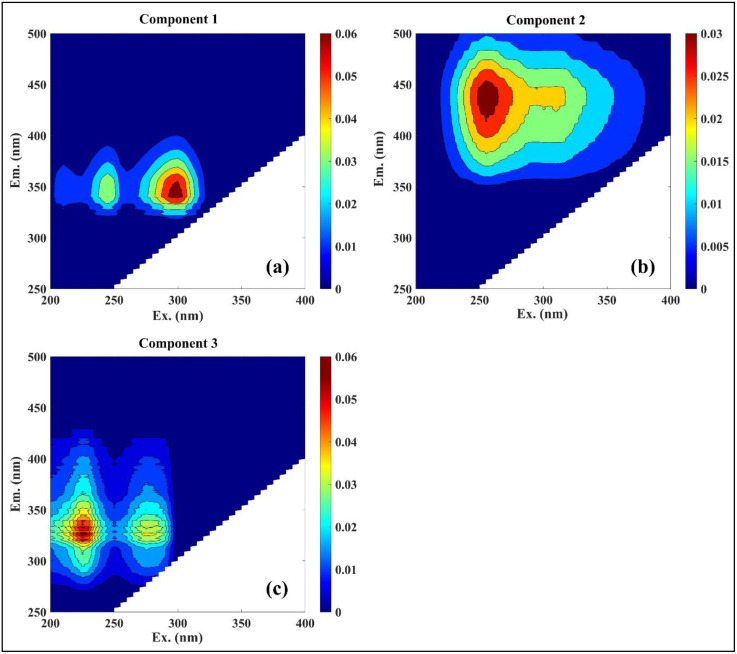
fDOM components in the fixed monitoring point identified by PARAFAC modelling: (a, c) protein-like and (b) fulvic acid (C-like).

Parallel factor analysis (PARAFAC) applied to excitation-emission matrices (EEMs) from 48 water samples in Kaptai Lake resolved three distinct fluorescent DOM (fDOM) components, reflecting diverse biogeochemical sources and optical characteristics. Component 1 (C1) exhibited two fluorescence peaks at Ex/Em = 300/346 nm and 245/346 nm (Fig 3a), corresponding to tryptophan-like and tyrosine-like proteinaceous compounds, respectively. This component is interpreted as a labile, low-molecular weight, autochthonous DOM fraction, typically associated with microbial or algal metabolic byproducts [57]. Its spectral positioning and intensity profile suggest high photoreactivity and rapid microbial turnover, consistent with prior observations in eutrophic and biologically productive systems [11,58]. Component 2 (C2) showed peaks at Ex/Em = 260/443 nm and 310/443 nm (Fig 3b), aligning with classic humic-like (C-like) and fulvic acid-like signatures. C2 is interpreted as a semi-labile, hydrophilic, low-aromatic, allochthonous DOM component, likely derived from terrestrial plant leachates and soil organic matter inputs [8,59].

**Fig 4:**
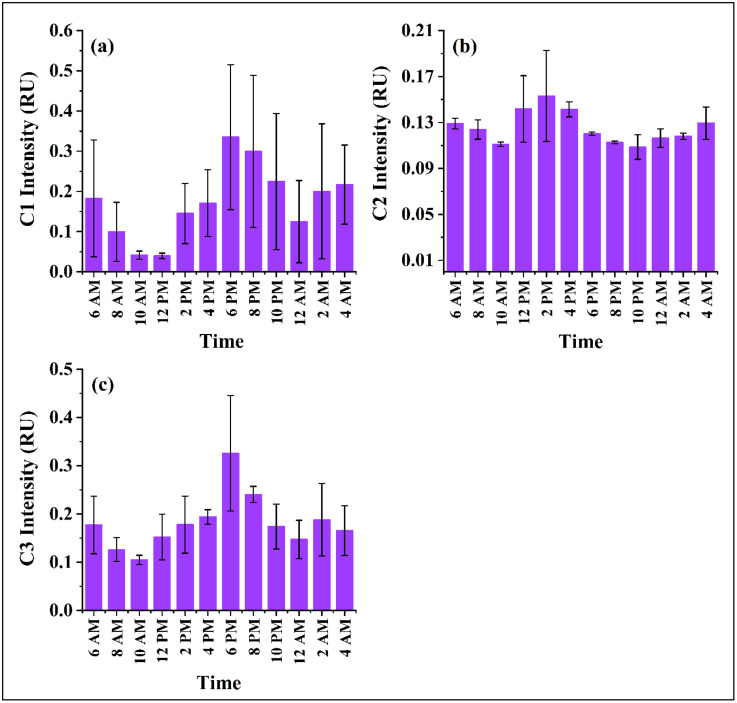
Diurnal variation of component intensity in the surface water of Kaptai Lake.

Its persistent fluorescence signals, even at high solar exposure, highlight its structural resilience and longer residence time in surface waters. Component 3 (C3) featured excitation-emission maxima at 230/338–350 nm (Peak Tuv) and 280/338–346 nm (Peak T) (Fig 3c), indicative of protein-like, aromatic, and photosensitive fDOM species. Unlike C1, C3 appears to be influenced by anthropogenic sources, including urban sewage discharge and effluent-related inputs, supported by literature connecting similar fluorescence signals to human wastewater [13,57,60]. Quantitatively, the intensity ranges for C1, C2, and C3 were 0.02–0.62 RU (0.17 ± 0.04), 0.09–0.19 RU (0.13 ± 0.005), and 0.09–0.53 RU (0.17 ± 0.02), respectively (Fig 4).

Temporal dynamics revealed distinct diel fluctuations in fDOM intensities, strongly tied to solar exposure and microbial activity. Protein-like components (C1 and C3) exhibited similar diurnal trends, with maximum intensities occurring in the late afternoon (4:00–6:00 PM), likely due to DOM accumulation from daytime microbial activity. Minimum intensities were recorded from mid-morning to mid-day (10:00 AM–12:00 PM), coinciding with peak solar radiation and photodegradation rates [57] (Fig 4a, c). In contrast, the fulvic-like component C2 displayed a bimodal pattern, with elevated intensities during early dawn (4:00–6:00 AM) and mid-afternoon (12:00–4:00 PM). These fluctuations may reflect both nocturnal microbial respiration and daytime thermal stimulation of microbial degradation, processes known to liberate fluorophores from organic substrates (Fig 4b). The observed diel behavior highlights the dynamic coupling between biological, photochemical, and thermal processes regulating DOM reactivity in tropical freshwater systems.

**Fig 5:**
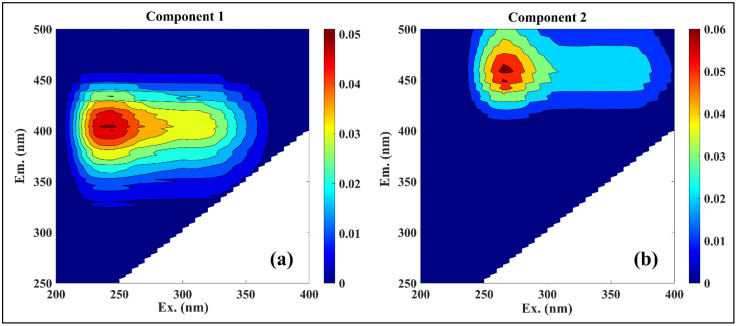
fDOM components in the spatial distribution samples identified by PARAFAC modelling: (a) fulvic acid (M-like) and (b) fulvic acid (C-like).

The spatial variability of fDOM components, derived from a complementary PARAFAC model, underscores the influence of both natural and anthropogenic inputs across the lake. In the spatial configuration, Component 1 (C1) exhibited peaks at Ex/Em = 310/404 nm (peak M) and 245/404 nm (peak A) (Fig 5a), characteristic of microbially reprocessed fulvic substances [61,62]. This form is interpreted as photodegradable yet labile DOM, primarily derived from algal assimilation and in situ microbial transformation processes [63]. Component 2 (C2) was spectrally assigned to Ex/Em = 265/460 nm and 370/460 nm, representing a more aromatic, terrestrial C-like fulvic acid signal (Fig 5b). This component was consistently associated with allochthonous input, likely from catchment-derived soil OM and leaf litter, and is characterized by moderate hydrophilicity and semi-lability [8,55,59]. C1 and C2 intensities ranged from 0.027–0.15 RU (0.08 ± 0.01) and 0.06–0.14 RU (0.08 ± 0.01), respectively (S3 Fig.). Higher concentrations were observed in nearshore zones adjacent to Rangamati town, where sewage discharge, tourism-related waste, and increased nutrient inputs are likely to enhance fDOM production. Conversely, upland catchments near forested Rangamati hill tracts are likely to supply terrestrially derived OM through runoff and soil leaching. This spatial gradient underscores the dual influence of anthropogenic activity and natural landscape processes in shaping DOM composition in tropical reservoir systems.

### Optical indices and sources of DOM

**Fig 6:**
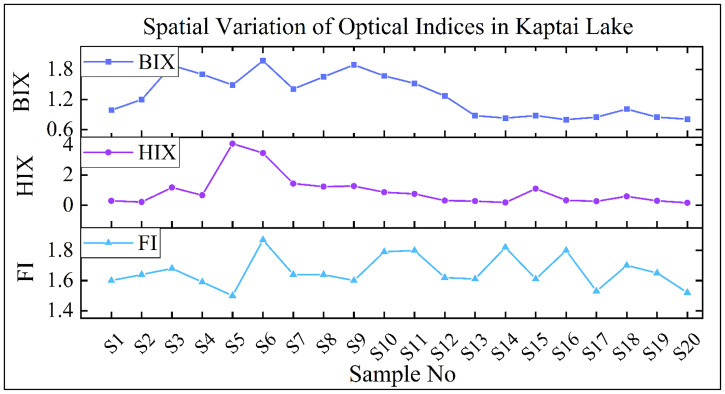
Distribution of DOM optical indices (FI, HIX, and BIX) across water samples from Kaptai Lake.

Fluorescence-based indices such as the Fluorescence Index (FI), Humification Index (HIX), and Biological Index (BIX) offer critical insight into the sources and transformation processes of dissolved organic matter (DOM) in aquatic ecosystems. FI is widely used to distinguish DOM origin microbial sources typically yield values between 1.7 and 1.9, while terrestrial sources fall within 1.3 to 1.4 [14,44]. In the current study, FI values in Kaptai Lake ranged from 1.48 to 1.87 (mean ± SD: 1.66 ± 0.11), indicating a clear predominance of autochthonous, microbially-derived DOM. This trend microbially dominated DOM with moderate nutrient enrichment and low terrestrial input has been observed in other Bangladeshi freshwater systems. For instance, fluorescence spectroscopy of the Ganges–Brahmaputra–Meghna basin has documented protein-like, autochthonous DOM components dominating during monsoonal periods [17]. The spatial distribution of FI, as depicted in Fig 6, shows sharp fluctuations, with peaks near samples S6, S11, and S16 suggesting localized hotspots of microbial productivity, possibly due to hydrological stagnation or point-source nutrient inputs. In contrast, lower values (e.g., S5, S15) may reflect occasional terrestrial influence or dilution from inflow zones. The HIX values, ranging from 0.16 to 4.08 (mean: 0.83 ± 0.99), are consistently below the threshold (HIX < 4) indicative of microbially-derived DOM and low humification [18,45]. These low values confirm the prevalence of freshly produced DOM with minimal diagenetic alteration. Interestingly, the HIX peak observed around S5–S6 (up to 4.08) suggests a possible transient influx of allochthonous material, potentially driven by sediment resuspension or episodic runoff. The BIX values in Kaptai Lake, ranging from 0.80 to 1.97 (mean: 1.20 ± 0.42), further affirm the dominance of biological origin DOM. According to [46], BIX values >1 denote strong bacterial activity and freshly produced DOM. In this study, nearly all stations exceed the 1.0 threshold, with several (e.g., S5–S7, S9–S10) reaching values above 1.6 reflecting intense microbial production and rapid DOM turnover in those zones. Comparable fluorescence-based studies in the Ganges, Brahmaputra, Meghna River waters have reported elevated BIX values above 1 during periods of heightened biological activity in surface waters (e.g., during monsoon and post-monsoon seasons) [18].

Beyond optical fluorescence, absorbance-based indices such as E_250_:E_365_, Specific UV Absorbance at 254 nm (SUVA_254_), and the Slope Ratio (SR) (S_275–295_/S_350–400_) serve as valuable indicators of DOM molecular composition, aromaticity, and size fractionation. The E_250_:E_365_ values in Kaptai Lake varied from 3.14 to 4.97 (mean ± SD: 4.15 ± 0.67), while SR ranged from 1.69 to 2.94 (mean: 2.05 ± 0.59), suggesting the dominance of low molecular weight DOM fractions. Such ratios typically indicate freshly generated microbial DOM and reduced terrestrial contribution, which aligns with the high BIX and low HIX trends observed. SUVA_254_, calculated as UV absorbance at 254 nm normalized to DOC concentration, is widely used to estimate DOM aromaticity and molecular complexity. In Kaptai Lake, SUVA_254_ ranged from 5.15 to 6.36 LmgC^-1^m^-1^ (mean: 5.71 ± 0.47), hovering near the upper limit of typical surface water values. While high SUVA_254_ values often suggest terrestrial-derived aromatic DOM [64], the simultaneous presence of low HIX and high FI suggests that in this case, the elevated values may be attributed to microbial DOM containing reactive unsaturated structures or partially oxidized aromatic intermediates [46]. Taken together, the suite of optical and absorbance indices reveals that DOM in Kaptai Lake is predominantly microbially derived, of low molecular weight, and rapidly cycling driven by internal productivity, with intermittent influence from terrestrial or sedimentary inputs. The combined analysis of FI, HIX, BIX, SUVA_254_, and E_250_:E_365_ enables a robust understanding of the biogeochemical footprint and source character of DOM in this ecologically vital lake system. These findings are consistent with several studies in freshwater and estuarine systems, where elevated E₂₅₀:E₃₆₅ and SR values, alongside high BIX and low HIX, have been reliably used to indicate freshly produced, microbially derived, low molecular weight DOM with minimal terrestrial influence [42,65].

### Diurnal variation in pCO_2_ and CO_2_ flux

**Fig 7:**
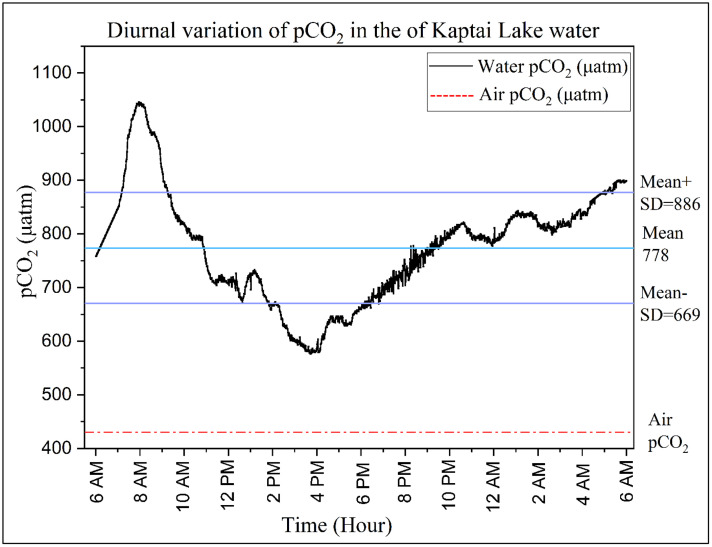
Diurnal variation of *p*CO_2_ in the surface water of Kaptai Lake.

The partial pressure of CO₂ (pCO_2_) in Kaptai Lake exhibited a marked diurnal cycle during the observation period, ranging from 577 to 1045 µatm, with a mean ± standard deviation of 778 ± 108 µatm (Fig 7). The water consistently shows pCO_2_ values far above the atmospheric baseline (~430 µatm), as indicated by the red dashed line in Fig 7. This strong supersaturation highlights Kaptai Lake as a net CO_2_ source to the atmosphere. Diurnal variation was evident, with the highest pCO_2_ concentrations occurring during early morning hours (4:00–8:00 AM), reaching values near 1045 µatm well above the atmospheric pCO_2_ levels. This peak corresponds to the accumulation of respired CO_2_ overnight, as photosynthesis halts in the absence of light, while microbial and macrofaunal respiration continues [66,67]. Moreover, lower nocturnal water temperatures increase gas solubility, further enhancing CO_2_ concentration [68]. As sunlight intensifies after sunrise, photosynthetic uptake by phytoplankton and aquatic vegetation rapidly draws down CO_2_, resulting in the lowest pCO_2_ values (as low as 577 µatm) during the afternoon (2:00–6:00 PM), as visualized by the sharp mid-day trough in Fig 7. This inverse relationship between light-driven photosynthesis and CO_2_ levels is consistent with diel carbon dynamics described in similar lentic systems [16,69]. Although water temperatures increase during daylight, potentially reducing CO_2_ solubility and accelerating OM decomposition [70], the minor diurnal temperature variation observed in Kaptai Lake likely limits its impact on respiration-driven CO_2_ release [16]. Notably, some irregular spikes in pCO_2_ particularly during post-midnight to dawn may correspond to rainfall events, which can introduce CO_2_ rich runoff or disturb the equilibrium at the air–water interface.

As pCO_2_ serves as a proxy for aquatic carbon dynamics, quantifying the flux of CO_2_ across the air-water interface is critical. The diffusive flux (FCO_2_) from Kaptai Lake ranged from 45 to 56 mmol CO_2_ m^−2^ d^−1^, averaging 50 ± 5 mmol CO_2_ m^−2^ d^−1^. These values, consistently positive, indicate a net efflux of CO_2_ from the lake surface to the atmosphere, classifying Kaptai Lake as a regional CO_2_ source rather than a sink.

A comparison with other lakes worldwide reveals distinct characteristics in terms of CO_2_ flux (Table 2). Whether a lake functions as a carbon sink or source depends on various natural and anthropogenic factors. For instance, while Brazilian tropical systems like Lake Tupe (3187 ± 29 µatm, 77.45 mmol CO_2_ m^−2^ d^−1^) and Lake Calado (1121 ± 22 µatm, 44.45 mmol CO_2_ m^−2^ d^−1^) exhibit higher or comparable CO_2_ fluxes, Kaptai flux rate surpasses that of many temperate and boreal systems. Australian lakes such as Lake Albert (448 ± 93 µatm) and Lake Alexandria (485 ± 143 µatm during drought, declining post-drought) recorded much lower pCO_2_ values and correspondingly lower CO_2_ fluxes. Similarly, Swedish lakes like Parsen, Venasjön, and Ljusvattentjärn, though varying in size and catchment area, consistently exhibit lower FCO_2_ (ranging from ~10–76 mmol CO_2_ m^−2^ d^−1^), with most well below Kaptai Lake’s average. Therefore, in a global context, Kaptai Lake’s FCO_2_ places it in a mid-to-high range, reflecting its large surface area (688 km^2^), substantial catchment (11,200 km^2^), and influence from both natural and anthropogenic inputs. Key drivers include terrestrial organic matter influx, seasonal precipitation, and watershed activities such as dam operation, erosion, and land use changes [77]. According to [32], such variables along with lake morphometry and productivity play decisive roles in regulating carbon dynamics in inland waters.

**Table 2:** Daily average *p*CO_2_ of water and *F*CO_2_ of some lakes worldwide.

Region	Area (km^2^)	Catchment Area (km^2^)	*p*CO_2_ (µatm)	*F*CO_2_ (mmol CO_2_ m^−2^d^−1^)	References
Kaptai Lake	688	11,200	831 ± 135	45-56	This study
Lake Alexandria, Australia	650	1,061,469	485 ± 143	0.30-7.0 (drought period) −16.40 and 0.90 (post-drought)	[71]
Lake Albert, Australia	170	280	448 ± 93		[71]
High-Latitude Lakes, Sweden (14 lakes)	–	–	–	0.062-1.55	[30]
Ross Barnett Reservoir, North America	134	22,714		22.46-26.78	[72]
Lochaber Lake, Canada	13.5			14.77 ± 1.99	[73]
Lake Tupe, Brazil	–	–	3187 ± 29	77.45	[29]
Lake Calado, Brazil	2.12	–	1121 ± 22	44.45	[29]
Lake Erie, North America	25700			12.09-36.28	[74]
Venasjön Lake, Sweden	0.68	170	–	76 ± 26.2	[75]
Parsen Lake, Sweden	0.13	1.40	–	24.3 ± 11.4	[75]
Ljusvattentjärn Lake, Sweden	0.017	0.89	–	9.9 ± 3.2	[75]
Lake Tämnaren, Sweden	38	1,200	–	19.008	[76]

### Factors regulating pCO_2_ dynamics

**Fig 8:**
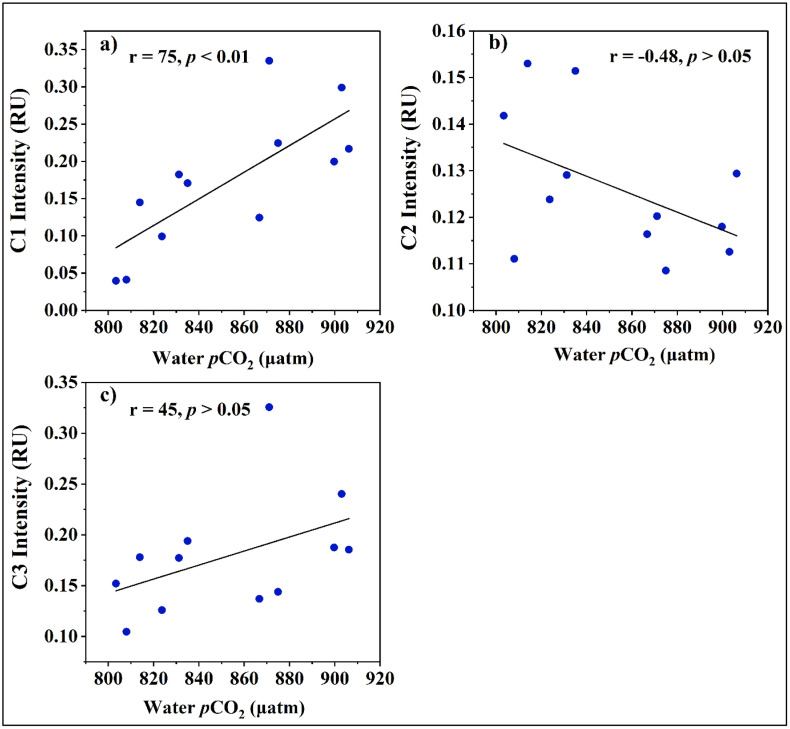
Relationship between water *p*CO_2_ and fDOM component intensity in Kaptai Lake water.

The partial pressure of CO_2_ (pCO_2_) in Kaptai Lake exhibited strong sensitivity to key biogeochemical parameters, notably dissolved oxygen (DO), fluorescent dissolved organic matter (fDOM), and fluorescence-derived DOM components (S4 Fig, Fig 8). A significant negative correlation was observed between pCO_2_ and DO (r^2^ = 0.64, p < 0.001; S4a Fig), indicating that lower oxygen concentrations correspond with elevated CO₂ levels. This inverse relationship reflects the metabolic balance of aquatic ecosystems: during periods of oxygen depletion typically driven by microbial and faunal respiration CO_2_ accumulates as a metabolic byproduct, especially when photosynthetic oxygen production is minimal. This highlights the diel oxygen–carbon coupling in stratified or biologically active systems like Kaptai Lake [78]. In contrast, a weaker but statistically significant positive correlation was found between pCO_2_ and fDOM intensity (r^2^ = 0.12, p < 0.001) (S4b Fig). This relationship suggests that fluorescent DOM, often derived from microbial or terrestrial sources, contributes to the carbon pool as it undergoes decomposition via microbial respiration or photochemical processes. Though the strength of this correlation is modest, it underscores the potential for fDOM to act as a substrate for microbial mineralization, releasing CO_2_ into the water column.

Further insight was gained from fluorescence component analysis. The protein-like components C1 and C3 showed positive correlations with pCO_2_ (C1: r = 0.75, p < 0.01; C3: r = 0.45, p > 0.05; Figs 8a and 8c). These components, typically representative of tryptophan-like and tyrosine-like DOM, are often associated with microbial exudates and freshly produced autochthonous material. Their strong association with pCO_2_ supports the hypothesis that labile, nitrogen-rich DOM enhances microbial respiration, thereby increasing CO_2_ production in the water column [79]. Conversely, a moderate negative correlation was found between pCO_2_ and the humic-like (fulvic acid-like) component (C-like; r = 0.48, p > 0.05; Fig 8b). Humic-like (fulvic acid-like) DOM fractions are typically more aromatic, structurally complex, and nutrient-poor, which makes them less bioavailable and resistant to microbial degradation compared to protein-like components [80]. As a result, their contribution to short-term CO_2_ generation is limited, often leading to weak or negative correlations with pCO_2_ in aquatic systems. Previous studies have reported that increasing proportions of refractory, humic-rich DOM are associated with reduced microbial respiration and lower CO_2_ production, as microbes preferentially utilize labile, nitrogen-rich substrates [4,81]. This persistence of fulvic substances reflects a more stable DOM pool that resists rapid mineralization, thereby buffering CO_2_ accumulation in lake waters [14]. Collectively, these results suggest that temporal and spatial variation in pCO_2_ in Kaptai Lake is driven by a complex interplay between oxygen availability, DOM composition, and microbial activity. Protein-like DOM components fuel CO_2_ production, whereas fulvic-rich fractions appear to buffer CO_2_ accumulation, potentially stabilizing lake carbon emissions [32,78].

## Conclusion

The physicochemical parameters of Kaptai Lake indicated that the water quality mostly met national standards. Fluorescence spectroscopy provided valuable insight into the pollution status and sources of DOM in the lake, highlighting subtle changes not captured by conventional analyses. Protein-like and fulvic acid-like substances were detected, though their intensities remained low, suggesting limited organic pollution across most sites. FI, HIX, BIX, E_250_:E_365_, SR, and SUVA_254_ values suggested that fDOM components were primarily microbially derived, with low aromaticity. Spatially, higher intensities near Rangamati town point to localized pollution inputs, likely from nearby hotels, resorts, and urban runoff. These patterns indicate that human activities along the shoreline are influencing the composition and reactivity of DOM in certain areas. Additionally, the link between DOM and CO_2_ levels indicates that variations in DOM quality and origin may influence lake-atmosphere CO_2_ exchange. As the largest man-made lake in Bangladesh, Kaptai Lake remains an important freshwater body and a potential contributor to regional atmospheric CO_2_, especially under ongoing land-use and climate changes. These findings emphasize the need for integrated monitoring efforts combining chemical, optical, and ecological indicators to support informed management of this vital aquatic system.

## Supporting information

S1 File Deciphering CO2 flux and fluorescent DOM origins in the carbon cycle of Kaptai Lake. (DOCX)

## References

[1] Suman KH, Hossain MS, Salam MA, Rupok QSS, Haque MN. Production Trends, and Challenges for Biodiversity Conservation and Sustainable Fisheries Management of Kaptai Lake, the Largest Reservoir in Bangladesh. Asian Fish Sci. 2021;34(2). doi: 10.33997/j.afs.2021.34.2.004

[2] Carstea EM, Popa CL, Baker A, Bridgeman J. In situ fluorescence measurements of dissolved organic matter: A review. Sci Total Environ. 2020;699:134361. doi: 10.1016/j.scitotenv.2019.134361 31683216

[3] Pagano T, Bida M, Kenny J. Trends in Levels of Allochthonous Dissolved Organic Carbon in Natural Water: A Review of Potential Mechanisms under a Changing Climate. Water. 2014;6(10):2862–97. doi: 10.3390/w6102862

[4] Kellerman AM, Dittmar T, Kothawala DN, Tranvik LJ. Chemodiversity of dissolved organic matter in lakes driven by climate and hydrology. Nat Commun. 2014;5:3804. doi: 10.1038/ncomms4804 24787272

[5] Battin TJ, Luyssaert S, Kaplan LA, Aufdenkampe AK, Richter A, Tranvik LJ. The boundless carbon cycle. Nature Geosci. 2009;2(9):598–600. doi: 10.1038/ngeo618

[6] Bertilsson S, Jones JB. Supply of dissolved organic matter to aquatic ecosystems: autochthonous sources. In: Findlay SEG, Sinsabaugh RL, editors. Aquatic ecology. Academic Press; 2003. p. 3–24.

[7] Fuentes JL, Garbayo I, Cuaresma M, Montero Z, González-Del-Valle M, Vílchez C. Impact of Microalgae-Bacteria Interactions on the Production of Algal Biomass and Associated Compounds. Mar Drugs. 2016;14(5):100. doi: 10.3390/md14050100 27213407 PMC4882574

[8] Niloy NM, Haque MdM, Tareq SM. Characterization of dissolved organic matter at urban and industrial rainwater of Bangladesh by fluorescence spectroscopy and EEM-PARAFAC modeling. Environ Challenges. 2021;5:100250. doi: 10.1016/j.envc.2021.100250

[9] Ray R, Michaud E, Aller RC, Vantrepotte V, Gleixner G, Walcker R, et al. The sources and distribution of carbon (DOC, POC, DIC) in a mangrove dominated estuary (French Guiana, South America). Biogeochemistry. 2018;138(3):297–321. doi: 10.1007/s10533-018-0447-9

[10] Miah O, Roy A, Sakib AA, Niloy NM, Haque MM, Shammi M, et al. Diurnal and seasonal variations of pCO2 and fluorescent dissolved organic matter (FDOM) in different polluted lakes. Environ Sci Pollut Res Int. 2023;30(40):92720–35. doi: 10.1007/s11356-023-28878-y 37495806

[11] Coble PG. Characterization of marine and terrestrial DOM in seawater using excitation-emission matrix spectroscopy. Mar Chem. 1996;51(4):325–46. doi: 10.1016/0304-4203(95)00062-3

[12] Stedmon CA, Bro R. Characterizing dissolved organic matter fluorescence with parallel factor analysis: a tutorial. Limnol Oceanogr Methods. 2008;6(11):572–9. doi: 10.4319/lom.2008.6.572

[13] Mostofa KMG, Liu C, Vione D, Mottaleb MA, Ogawa H, Yoshioka T. Dissolved organic matter in natural waters. In: Environmental science and engineering. Springer; 2013. p. 1–137.

[14] McKnight DM, Boyer EW, Westerhoff PK, Doran PT, Kulbe T, Andersen DT. Spectrofluorometric characterization of dissolved organic matter for indication of precursor organic material and aromaticity. Limnol Oceanogr. 2001;46(1):38–48. doi: 10.4319/lo.2001.46.1.0038

[15] Hassan MA, Shammi M, Tareq SM. The deciphering of microplastics-derived fluorescent dissolved organic matter in urban lakes, canals, and rivers using parallel factor analysis modeling and mimic experiment. Water Environ Res. 2024;96(5):1–13. doi: 10.1002/wer.11041 38797514

[16] Haque MdM, Begum MS, Nayna OK, Tareq SM, Park J. Seasonal shifts in diurnal variations of pCO2 and O2 in the lower Ganges River. Limnol Oceanogr Letters. 2022;7(3):191–201. doi: 10.1002/lol2.10246

[17] Niloy NM, Haque MM, Tareq SM. Temporal changes in hydrochemistry and DOM characteristics of the Brahmaputra River: implication to the seasonality of water quality. Environ Sci Pollut Res Int. 2022;29(23):35165–78. doi: 10.1007/s11356-022-18618-z 35044604

[18] Niloy NM, Haque MdM, Tareq SM. Characteristics, Sources, and Seasonal Variability of Dissolved Organic Matter (DOM) in the Ganges River, Bangladesh. Environ Process. 2021;8(2):593–613. doi: 10.1007/s40710-021-00499-y

[19] Parvin F, Hassan MdA, Tareq SM. Risk assessment of microplastic pollution in urban lakes and peripheral Rivers of Dhaka, Bangladesh. J Hazard Mater Adv. 2022;8:100187. doi: 10.1016/j.hazadv.2022.100187

[20] Panchenko MV, Domysheva VM, Pestunov DA, Sakirko MV, Shamrin AM, Shmargunov VP. Carbon dioxide in the atmosphere-water system and biogenic elements in the littoral zone of Lake Baikal during period 2004–2018. J Great Lakes Res. 2020;46(1):85–94. doi: 10.1016/j.jglr.2019.10.016

[21] Yaacob NFF, Mat Yazid MR, Abdul Maulud KN, Ahmad Basri NE. A Review of the Measurement Method, Analysis and Implementation Policy of Carbon Dioxide Emission from Transportation. Sustainability. 2020;12(14):5873. doi: 10.3390/su12145873

[22] Cory RM, Kling GW. Interactions between sunlight and microorganisms influence dissolved organic matter degradation along the aquatic continuum. Limnol Oceanogr Letters. 2018;3(3):102–16. doi: 10.1002/lol2.10060

[23] Xenopoulos MA, Barnes RT, Boodoo KS, Butman D, Catalán N, D’Amario SC, et al. How humans alter dissolved organic matter composition in freshwater: relevance for the Earth’s biogeochemistry. Biogeochemistry. 2021;154(2):323–48. doi: 10.1007/s10533-021-00753-3

[24] Panneer Selvam B, Natchimuthu S, Arunachalam L, Bastviken D. Methane and carbon dioxide emissions from inland waters in India - implications for large scale greenhouse gas balances. Glob Chang Biol. 2014;20(11):3397–407. doi: 10.1111/gcb.12575 24623552

[25] Jarvie HP, Neal C, Leach DV, Ryland GP, House WA, Robson AJ. Major ion concentrations and the inorganic carbon chemistry of the Humber rivers. Sci Total Environ. 1997;194–195:285–302. doi: 10.1016/s0048-9697(96)05371-5

[26] Sadro S, Melack JM. The Effect of an Extreme Rain Event on the Biogeochemistry and Ecosystem Metabolism of an Oligotrophic High-Elevation Lake. Arct Antarct Alp Res. 2012;44(2):222–31. doi: 10.1657/1938-4246-44.2.222

[27] Gudasz C, Bastviken D, Steger K, Premke K, Sobek S, Tranvik LJ. Erratum: Temperature-controlled organic carbon mineralization in lake sediments. Nature. 2010;466(7310):1134–1134. doi: 10.1038/nature09383 20651689

[28] Romera-Castillo C, Letscher RT, Hansell DA. New nutrients exert fundamental control on dissolved organic carbon accumulation in the surface Atlantic Ocean. Proc Natl Acad Sci U S A. 2016;113(38):10497–502. doi: 10.1073/pnas.1605344113 27582464 PMC5035860

[29] Almeida FV, Guimarães JR, Jardim WF. Measuring the CO2 flux at the air/water interface in lakes using flow injection analysis. J Environ Monit. 2001;3(3):317–21. doi: 10.1039/b010065j 11432270

[30] Chengxin F, Weiping H, Ford PW, Yuwei C, Wenchuan Q, Lu Z. Carbon dioxide partial pressure and carbon fluxes of air-water interface in Taihu Lake, China. Chin J Ocean Limnol. 2005;23(1):29–38. doi: 10.1007/bf02845140

[31] Kelly CA, Fee E, Ramlal PS, Rudd JWM, Hesslein RH, Anema C, et al. Natural variability of carbon dioxide and net epilimnetic production in the surface waters of boreal lakes of different sizes. Limnology & Oceanography. 2001;46(5):1054–64. doi: 10.4319/lo.2001.46.5.1054

[32] Regnier P, Friedlingstein P, Ciais P, Mackenzie FT, Gruber N, Janssens IA, et al. Anthropogenic perturbation of the carbon fluxes from land to ocean. Nature Geosci. 2013;6(8):597–607. doi: 10.1038/ngeo1830

[33] Hoque MM, Kabir MR, Nahar N. Water quality status of Dhanmondi Lake in Dhaka City: a multivariate statistical approach. J Water Resour Prot. 2020;12(4):259–75. doi: 10.4236/jwarp.2020.124015

[34] Karmakar S, Sirajul Haque SM, Mozaffar Hossain M, Shafiq M. Water quality of Kaptai reservoir in Chittagong Hill Tracts of Bangladesh. J Forest Res. 2011;22(1):87–92. doi: 10.1007/s11676-011-0131-6

[35] Johnson MS, Billett MF, Dinsmore KJ, Wallin M, Dyson KE, Jassal RS. Direct and continuous measurement of dissolved carbon dioxide in freshwater aquatic systems—method and applications. Ecohydrology. 2009;3(1):68–78. doi: 10.1002/eco.95

[36] Murphy KR, Stedmon CA, Wenig P, Bro R. OpenFluor—an online spectral library of fluorescence excitation-emission matrices (EEMs) for the identification and comparison of dissolved organic matter components. Anal Chim Acta. 2014;803:21–33. doi: 10.1016/j.aca.2013.10.006

[37] Nayna OK, Begum MS, Ran L, Park J-H. Improving Carbonate Equilibria-Based Estimation of pCO2 in Anthropogenically Impacted River Systems. Front Earth Sci. 2021;9. doi: 10.3389/feart.2021.778215

[38] Lauerwald R, Laruelle GG, Hartmann J, Ciais P, Regnier PAG. Spatial patterns in CO2 evasion from the global river network. Global Biogeochemical Cycles. 2015;29(5):534–54. doi: 10.1002/2014gb004941

[39] Raymond PA, Zappa CJ, Butman D, Bott TL, Potter J, Mulholland P, et al. Scaling the gas transfer velocity and hydraulic geometry in streams and small rivers. Limn Fluids and Environments. 2012;2(1):41–53. doi: 10.1215/21573689-1597669

[40] Cook S, Peacock M, Evans CD, Page SE, Whelan MJ, Gauci V, et al. Quantifying tropical peatland dissolved organic carbon (DOC) using UV-visible spectroscopy. Water Res. 2017;115:229–35. doi: 10.1016/j.watres.2017.02.059 28284089

[41] Weishaar JL, Aiken GR, Bergamaschi BA, Fram MS, Fujii R, Mopper K. Evaluation of specific ultraviolet absorbance as an indicator of the chemical composition and reactivity of dissolved organic carbon. Environ Sci Technol. 2003;37(20):4702–8. doi: 10.1021/es030360x 14594381

[42] Helms JR, Stubbins A, Ritchie JD, Minor EC, Kieber DJ, Mopper K. Absorption spectral slopes and slope ratios as indicators of molecular weight, source, and photobleaching of chromophoric dissolved organic matter. Limnology & Oceanography. 2008;53(3):955–69. doi: 10.4319/lo.2008.53.3.0955

[43] John RH, Stubbins A, Ritchie JD, Minor EC, Kieber DJ, Mopper K. Erratum: absorption spectral slopes and slope ratios as indicators of molecular weight, source, and photobleaching of chromophoric dissolved organic matter. Limnology and Oceanography. 2009;54(3):1023.

[44] Cory RM, McKnight DM. Fluorescence spectroscopy reveals the composition and photochemical reactivity of dissolved organic matter in an alpine stream. Biogeochemistry. 2005;74(2):213–39. doi: 10.1007/s10533-004-3464-9

[45] Ohno T. Fluorescence inner-filtering correction for determining the humification index of dissolved organic matter. Environ Sci Technol. 2002;36(4):742–6. doi: 10.1021/es0155276 11878392

[46] Huguet A, Vacher L, Relexans S, Saubusse S, Froidefond JM, Parlanti E. Properties of fluorescent dissolved organic matter in the Gironde Estuary. Org Geochem. 2009;40(6):706–19. doi: 10.1016/j.orggeochem.2009.03.002

[47] Tushar MAN, Hossain A, Hossen M, Siddique MAB, Hasan MM, Rahman MS. Spatiotemporal assessment of water quality and quantity of the Kaptai Lake at Rangamati, Bangladesh. Dhaka Univ J Earth Environ Sci. 2024;12(1):41–52. doi: 10.3329/dujees.v12i1.77581

[48] Ali MK, Jubaer A, Zafar MT, Talukder MZI. Physicochemical assessment of Dhanmondi Lake water in Dhaka city, Bangladesh. Eur J Chem. 2022;13(2):126–31. doi: 10.5155/eurjchem.13.2.126-131.2304

[49] Khondker M, Alfasane MA, Islam MS, Bhuiyan MAH, Gani MA. Limnology of Lake Bogakain, Bandarban, Bangladesh. Bangladesh J Bot. 1970;39(2):153–9. doi: 10.3329/bjb.v39i2.7301

[50] Mosaddequr Rahman M, Abul Bashar M, Farhana Z, Yeamin Hossain M. Temporal variation of physicochemical parameters in Kaptai Lake, Bangladesh. World J Fish Mar Sci. 2014;6(5):475–8. doi: 10.5829/idosi.wjfms.2014.06.05.8696

[51] Chowdhury DA, Jamal M, Ahmed U, Helal M. Physico-chemical characterization of Kaptai Lake and Foy’s Lake water quality parameters in Chittagong, Bangladesh. Am J Pure Appl Biosci. 2019;1(6):49–58.

[52] Barua R, Barua S, Tuz-Zohora F, Mutsuddi R, Uddin MS, Hasegawa H, et al. Bacteriological and Physicochemical Characteristics of Kaptai Lake Water in Terms of Public Health Significance. Int J Sci Res Environ Sci. 2016;4(2):31–9. doi: 10.12983/ijsres-2016-p0031-0039

[53] Niloy NM, Habib SA, Islam MI, Haque MM, Shammi M, Tareq SM. Distribution, characteristics and fate of fluorescent dissolved organic matter (FDOM) in the Bay of Bengal. Mar Pollut Bull. 2023;195:115467. doi: 10.1016/j.marpolbul.2023.115467 37659388

[54] Cory RM, Kaplan LA. Biological lability of streamwater fluorescent dissolved organic matter. Limnol Oceanogr. 2012;57(5):1347–60. doi: 10.4319/lo.2012.57.5.1347

[55] Cory RM, McKnight DM, Chin Y, Miller P, Jaros CL. Chemical characteristics of fulvic acids from Arctic surface waters: Microbial contributions and photochemical transformations. J Geophys Res. 2007;112(G4). doi: 10.1029/2006jg000343

[56] Gao S-J, Zhao C, Shi Z-H, Zhong J, Liu J-G, Li J-Q. Spectroscopic Characteristics of Dissolved Organic Matter in Afforestation Forest Soil of Miyun District, Beijing. J Anal Methods Chem. 2016;2016:1480857. doi: 10.1155/2016/1480857 27433371 PMC4940562

[57] Murphy KR, Stedmon CA, Waite TD, Ruiz GM. Distinguishing between terrestrial and autochthonous organic matter sources in marine environments using fluorescence spectroscopy. Mar Chem. 2008;108(1–2):40–58. doi: 10.1016/j.marchem.2007.10.003

[58] Coble PG, Del Castillo CE, Avril B. Distribution and optical properties of CDOM in the Arabian Sea during the 1995 Southwest Monsoon. Deep Sea Res Part II Top Stud Oceanogr. 1998;45(10–11):2195–223. doi: 10.1016/s0967-0645(98)00068-x

[59] Mostofa KMG, Yoshioka T, Konohira E, Tanoue E, Hayakawa K, Takahashi M. Three-dimensional fluorescence as a tool for investigating the dynamics of dissolved organic matter in the Lake Biwa watershed. Limnology. 2005;6(2):101–15. doi: 10.1007/s10201-005-0149-6

[60] Mostofa KMG, Wu F, Liu C-Q, Fang WL, Yuan J, Ying WL, et al. Characterization of Nanming River (southwestern China) sewerage-impacted pollution using an excitation-emission matrix and PARAFAC. Limnology. 2009;11(3):217–31. doi: 10.1007/s10201-009-0306-4

[61] Fu P, Mostofa KMG, Wu F, Liu C-Q, Li W, Liao H, et al. Excitation-emission matrix characterization of dissolved organic matter sources in two eutrophic lakes (Southwestern China Plateau). Geochem J. 2010;44(2):99–112. doi: 10.2343/geochemj.1.0047

[62] Wang Z-G, Liu W-Q, Zhao N-J, Li H-B, Zhang Y-J, Si-Ma W-C, et al. Composition analysis of colored dissolved organic matter in Taihu Lake based on three dimension excitation-emission fluorescence matrix and PARAFAC model, and the potential application in water quality monitoring. J Environ Sci. 2007;19(7):787–91. doi: 10.1016/s1001-0742(07)60132-6 17966864

[63] Aoki S, Ohara S, Kimura K, Mizuguchi H, Fuse Y, Yamada E. Characterization of fluorophores released from three kinds of lake phytoplankton using gel chromatography and fluorescence spectrophotometry. Anal Sci. 2008;24(11):1461–7. doi: 10.2116/analsci.24.1461 18997376

[64] Jaffé R, McKnight D, Maie N, Cory R, McDowell WH, Campbell JL. Spatial and temporal variations in DOM composition in ecosystems: The importance of long‐term monitoring of optical properties. J Geophys Res. 2008;113(G4). doi: 10.1029/2008jg000683

[65] Yamashita Y, Scinto LJ, Maie N, Jaffé R. Dissolved Organic Matter Characteristics Across a Subtropical Wetland’s Landscape: Application of Optical Properties in the Assessment of Environmental Dynamics. Ecosystems. 2010;13(7):1006–19. doi: 10.1007/s10021-010-9370-1

[66] Han B-P. Effect of photoinhibition on algal photosynthesis: a dynamic model. J Plankton Res. 2000;22(5):865–85. doi: 10.1093/plankt/22.5.865

[67] Markager S, Sand-Jensen K. Patterns of Night-Time Respiration in a Dense Phytoplankton Community Under a Natural Light Regime. J Ecol. 1989;77(1):49. doi: 10.2307/2260915

[68] Diurnal changes of stratification and photosynthesis in some tropical African waters. Proc R Soc Lond B. 1957;147(926):57–83. doi: 10.1098/rspb.1957.0036

[69] Gómez-Gener L, Rocher-Ros G, Battin T, Cohen MJ, Dalmagro HJ, Dinsmore KJ, et al. Global carbon dioxide efflux from rivers enhanced by high nocturnal emissions. Nat Geosci. 2021;14(5):289–94. doi: 10.1038/s41561-021-00722-3

[70] Peter H, Singer GA, Preiler C, Chifflard P, Steniczka G, Battin TJ. Scales and drivers of temporal pCO2 dynamics in an Alpine stream. J Geophys Res Biogeosci. 2014;119(6):1078–91. doi: 10.1002/2013jg002552

[71] Li S, Bush RT, Ward NJ, Sullivan LA, Dong F. Air-water CO2 outgassing in the Lower Lakes (Alexandrina and Albert, Australia) following a millennium drought. Sci Total Environ. 2016;542(Pt A):453–68. doi: 10.1016/j.scitotenv.2015.10.070 26520269

[72] Liu Y, Xu X, Zhang L, Liu Y, Li H. Temporal and spatial variations of air-water CO2 flux in the Ross Barnett Reservoir: a case study in Mississippi, USA. Water Air Soil Pollut. 2016;227(8):278.

[73] Spafford L, Risk D. Spatiotemporal variability of lake pCO2 and CO2 fluxes in a hemiboreal catchment. Limnol Oceanogr. 2018;63(S1):S149–66.

[74] Shao C, Chen J, Stepien CA, Tang J, Ouyang Z, Czajkowski KP. J Geophys Res Biogeosci. 2015;120(8):1582–604. doi: 10.1002/2015JG002973

[75] Rudberg D, Duc NT, Schenk J, Sieczko AK, Pajala G, Sawakuchi HO, et al. Diel Variability of CO2 Emissions From Northern Lakes. J Geophys Res Biogeosci. 2021;126(10). doi: 10.1029/2021jg006246

[76] Podgrajsek E, Sahlée E, Rutgersson A. Enhanced nighttime gas emissions from a lake. IOP Conf Ser Earth Environ Sci. 2016;35:012014. doi: 10.1088/1755-1315/35/1/012014

[77] Borges AV, Deirmendjian L, Bouillon S, Okello W, Lambert T, Roland FAE, et al. Greenhouse gas emissions from African lakes are no longer a blind spot. Sci Adv. 2022;8(25):eabi8716. doi: 10.1126/sciadv.abi8716 35749499 PMC9232103

[78] Raymond PA, Hartmann J, Lauerwald R, Sobek S, McDonald C, Hoover M, et al. Global carbon dioxide emissions from inland waters. Nature. 2013;503(7476):355–9. doi: 10.1038/nature12760 24256802

[79] Coble PG. Marine optical biogeochemistry: the chemistry of ocean color. Chem Rev. 2007;107(2):402–18. doi: 10.1021/cr050350+ 17256912

[80] Fellman JB, Hood E, Spencer RGM. Fluorescence spectroscopy opens new windows into dissolved organic matter dynamics in freshwater ecosystems: A review. Limnol Oceanograp. 2010;55(6):2452–62. doi: 10.4319/lo.2010.55.6.2452

[81] Koehler B, von Wachenfeldt E, Kothawala D, Tranvik LJ. Reactivity continuum of dissolved organic carbon decomposition in lake water. J Geophys Res. 2012;117(G1). doi: 10.1029/2011jg001793

