# Supplementary material for: Deciphering CO2 flux and fluorescent DOM origins in the carbon cycle of Kaptai Lake
Source: PLoS One. 2025 Nov 14;20(11):e0334646. doi: 10.1371/journal.pone.0334646 (PMC12617867; doi:10.1371/journal.pone.0334646)
Supplement: S1 File — (DOCX) [file pone.0334646.s001.docx]

**Deciphering CO_2_ flux and fluorescent DOM origins in the carbon cycle of Kaptai Lake**

Osman Miah^1^*, Rhidi Barma^1^, Md. Anamul Hassan^1^, Mashura Shammi^1^, Shafi M. Tareq^1^

^1^Hydrobiogeochemistry and Pollution Control Laboratory, Department of Environmental Sciences, Jahangirnagar University, Dhaka-1342, Bangladesh.

*Corresponding authors: [mashura926@juniv.edu](mailto:mashura926@juniv.edu) (MS); [osmanju75@gmail.com](mailto:osmanju75@gmail.com) (OM)

**Fig S1. Fluorescence intensity of the fluorophores in the 3D-EEM model in continuous monitoring in the Kaptai Lake.**

**Fig S2. Fluorescence intensity of the fluorophores in the 3D-EEM model in the spatial distribution.**

**
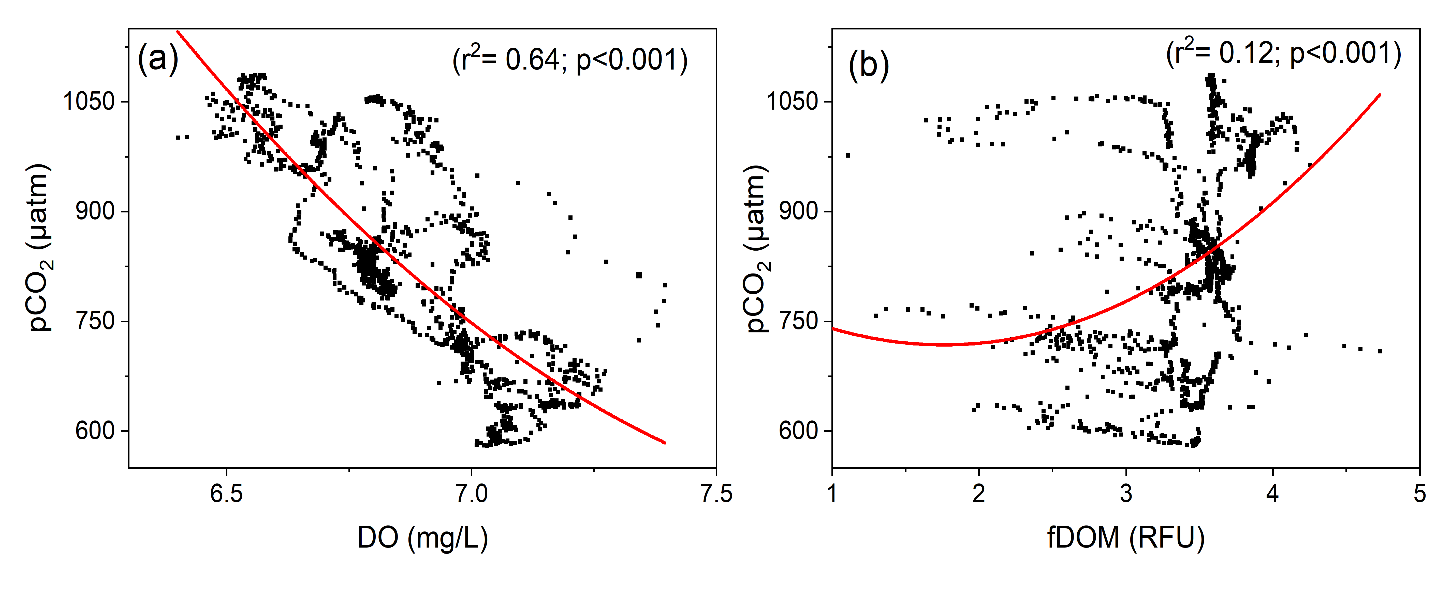
 Fig S3. Component intensity in spatial distribution in the surface water of Kaptai Lake.**

**Fig S4.** **Relation between *p*CO_2_ and DO (a) and fDOM and *p*CO_2_ in (b) of Kaptai Lake water.**
